# 1,4-Bis(2-diazo­acet­yl)piperazine

**DOI:** 10.1107/S1600536813018801

**Published:** 2013-07-13

**Authors:** Åsmund Kaupang, Carl Henrik Görbitz, Tore Bonge-Hansen

**Affiliations:** a Department of Chemistry, University of Oslo, PO Box 1033 Blindern, N-0315 Oslo, Norway

## Abstract

The asymmetric unit of the title compound, C_8_H_10_N_6_O_2_, contains one-half mol­ecule, which is completed by a crystallographic center of symmetry. The piperazine ring adopts a chair conformation. In the crystal, weak C—H⋯O inter­actions link the mol­ecules into layers parallel to the *bc* plane. The crystal packing also exhibits short N⋯N contacts of 3.0467 (16) Å between the terminal diazo N atoms from neighbouring mol­ecules.

## Related literature

For related structures in the Cambridge Structural Database (Version 5.34 of November 2012; Allen, 2002 ▶), see: Kaupang (2010 ▶); Kaupang *et al.* (2010 ▶, 2011 ▶); Aliev *et al.* (1980 ▶); Fitzgerald & Jensen (1978 ▶); Hope & Black (1972 ▶). For normal bond lengths in organic compounds, see: Allen *et al.* (1987 ▶). For synthetic details, see: Kaupang & Bonge-Hansen (2013 ▶); Kaupang (2010 ▶); Toma *et al.* (2007 ▶). For the synthesis of other diazo­acetamides with a 1,4-di­aza six-membered ring, see: Kaupang (2010 ▶); Mickelson *et al.* (1996 ▶). For the synthesis of other diazo­acetamides, see: Ouihia *et al.* (1993 ▶). For the Chemical Abstracts Service, see: American Chemical Society (2008 ▶). For graph-set notation for hydrogen-bonding patterns, see: Etter *et al.* (1990 ▶).
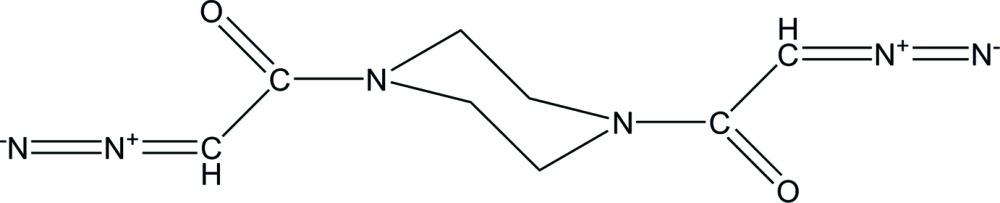


## Experimental

### 

#### Crystal data

C_8_H_10_N_6_O_2_
*M* *_r_* = 222.22
Monoclinic, 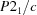

*a* = 4.0630 (7) Å
*b* = 9.0941 (15) Å
*c* = 13.230 (2) Å
β = 94.453 (2)°
*V* = 487.38 (14) Å^3^
*Z* = 2
Mo *K*α radiation
μ = 0.12 mm^−1^
*T* = 105 K
1.4 × 0.2 × 0.2 mm

#### Data collection

Bruker APEXII CCD diffractometer
Absorption correction: multi-scan (*SADABS*; Bruker, 2007 ▶) *T*_min_ = 0.859, *T*_max_ = 0.977
4255 measured reflections
1190 independent reflections
1013 reflections with *I* > 2σ(*I*)
*R*_int_ = 0.021

#### Refinement

*R*[*F* ^2^ > 2σ(*F* ^2^)] = 0.034
*wR*(*F* ^2^) = 0.090
*S* = 1.04
1190 reflections
82 parameters
3 restraints
H atoms treated by a mixture of independent and constrained refinement
Δρ_max_ = 0.30 e Å^−3^
Δρ_min_ = −0.22 e Å^−3^

### 

Data collection: *APEX2* (Bruker, 2007 ▶); cell refinement: *SAINT-Plus* (Bruker, 2007 ▶); data reduction: *SAINT-Plus*; program(s) used to solve structure: *SHELXTL* (Sheldrick, 2008 ▶); program(s) used to refine structure: *SHELXTL*; molecular graphics: *SHELXTL*; software used to prepare material for publication: *SHELXTL*.

## Supplementary Material

Crystal structure: contains datablock(s) I, New_Global_Publ_Block. DOI: 10.1107/S1600536813018801/cv5412sup1.cif

Structure factors: contains datablock(s) I. DOI: 10.1107/S1600536813018801/cv5412Isup2.hkl

Supplementary material file. DOI: 10.1107/S1600536813018801/cv5412Isup3.cml

Additional supplementary materials:  crystallographic information; 3D view; checkCIF report

## Figures and Tables

**Table 1:** Hydrogen-bond geometry (Å, °)

*D*—H⋯*A*	*D*—H	H⋯*A*	*D*⋯*A*	*D*—H⋯*A*
C2—H21⋯O1^i^	0.92 (1)	2.39 (1)	3.2219 (15)	151 (1)

Symmetry code: (i) 
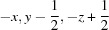
.

**Fig. 1.**
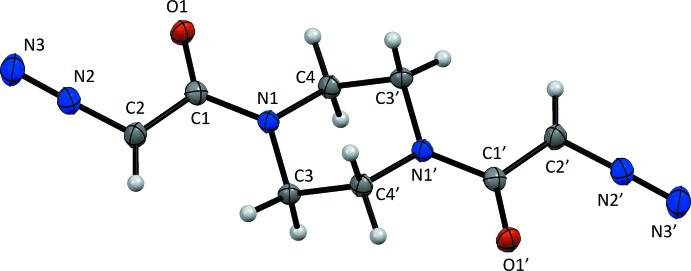
The molecular structure of (I) showing the atomic numbering and 50% probability displacement ellipsoids [symmetry code: (') -x, -y, 1 - z].

**Fig. 2.**
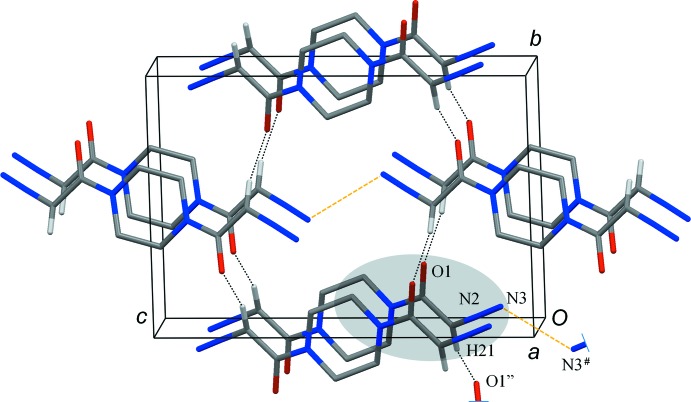
The unit cell and crystal packing of (I) viewed approximately along the a axis with the asymmetric unit covered by a grey ellipse. H atoms not involved in short intermolecular contacts (indicated by dotted lines) have been omitted for clarity. The molecules are connected by C21—H21···O1"(-x, y-1/2-y, 1/2-z) hydrogen bonds into two-dimensional sheets with C(4) chain and R44(26) ring motifs (for graph set notation, see Etter et al., 1990). An intermolecular N3···N3#(1-x,-y,-z) contact has been highlighted in orange.

## supplementary crystallographic information

### Comment

The *N,N'*-bis(2-diazoacetyl)piperazine (I) was prepared as part of a series of diazoacetamides (Kaupang *et al.*, 2010; 2011) used in the intramolecular C—H insertion reactions taking place upon thermolysis of their corresponding α-bromodiazoacetamides (Kaupang, 2010). The title compound was synthesized from *N,N'*-bis(2-bromoacetyl)piperazine following a procedure reported by Toma *et al.* (2007), modified to employ 1,1,3,3-tetramethylguanidine as the base instead of 1,8-diazabicyclo[5.4.0]undec-7-ene. No previous reports of this compound were found in the Chemical Abstracts Service (CAS; American Chemical Society, 2008).

In (I) (Fig. 1), the piperazine ring is in a normal chair conformation, with one half of the molecule constituting the asymmetric unit. In the Scheme the diazoacetyl group is illustrated in the normal way with C═N^+^ and N^+^═N^-^ double bonds, but sometimes a C—N single bond and a N≡N triple bond is used. N2—N3 = 1.1239 (14) Å is actually close in length to the triple bond in N_2_(*g*) = 1.0976 Å, but N2—C2 = 1.3099 (14) Å is clearly shorter than a normal N═N—C(*sp*^3^) single bond = 1.493 Å (Allen *et al.*, 1987), illustrating clearly the double bond nature.

As pointed out previously (Kaupang *et al.*, 2010), the ring N atoms have an amide rather than amine character and display an almost planar configuration [sum of C—N1—C angles 358.86 (15)°] due to the double bond character of N1—C1 = 1.3621 (13) Å. There are two interesting intermolecular interactions in the crystal packing (Fig. 2) - a C2—H21···O1 contact (Table 1) giving rise to chains and rings, and a 3.047 (14) Å N3···N3(1 - *x*,-*y*,-*z*) contact between the diazo groups with an associated N2—N3···N3 angle of 115.56 (10)° and N2—N3···N3—N2 torsion angle 180.0°. The diazoacetyl moiety is a relatively rare functional group occurring in 20 different organic molecules in the Cambridge Structural Database (CSD, Version 5.34 of November 2012; Allen 2002), and only three (Aliev *et al.*, 1980; Fitzgerald & Jensen, 1978; Hope & Black, 1972) participate in this type of interaction. Among more general compounds with a diazo group, 40 structures with 53 N···N distance < 3.5 Å were found in the CSD. Most contacts are within the 3.0 to 3.4 Å range. N—N···N angles have a wide distribution, but 45 are in the range 75 - 140° with average value 107°. A *trans* orientation for the N—N···N—N torsion angle is preferred; 36 out of 53 contacts have values in the range 180 ±20°. Almost all structures with N···N contacts < 3.2 Å fall into this group.

### Experimental

A solution of 4.0 mg of the title compound in 500 µ*L* of MeCN was placed in a vial measuring 30 × 6 mm and the open vial stored in the dark, exposed to air and at ambient temperature. Slow evaporation afforded yellow needles after 48 h. Crystals are rather fragile and easily fracture if cut with a scalpel. A rather long needle, 1.4 × 0.2 × 0.2 mm, was thus used for data collection.

### Refinement

H atoms bonded to C4 were positioned with idealized geometry and with fixed C—H distances at 0.99 Å, while positional coordinates were refined for H atoms bonded to C2 and C3, as too short intramolecular H···H distances resulted from putting these H atoms in theoretical positions. Distance restraints were imposed on the C2—H21 and C3—H31/H32 bonds utilizing *SHELX DFIX* 0.95 0.02 and *DFIX* 0.99 0.02 commands, respectively.

### Crystal data

**Table:**

C_8_H_10_N_6_O_2_	*F*(000) = 232
*M**_r_* = 222.22	*D*_x_ = 1.514 Mg m^−^^3^
Monoclinic, *P*2_1_/*c*	Melting point: 382 K
Hall symbol: -P 2ybc	Mo *K*α radiation, λ = 0.71073 Å
*a* = 4.0630 (7) Å	Cell parameters from 1727 reflections
*b* = 9.0941 (15) Å	θ = 2.7–28.7°
*c* = 13.230 (2) Å	µ = 0.12 mm^−^^1^
β = 94.453 (2)°	*T* = 105 K
*V* = 487.38 (14) Å^3^	Needle, yellow
*Z* = 2	1.4 × 0.2 × 0.2 mm

### Data collection

**Table:**

Bruker APEXII CCD diffractometer	1190 independent reflections
Radiation source: fine-focus sealed tube	1013 reflections with *I* > 2σ(*I*)
Graphite monochromator	*R*_int_ = 0.021
Detector resolution: 8.3 pixels mm^-1^	θ_max_ = 28.7°, θ_min_ = 2.7°
Sets of exposures each taken over 0.5° ω rotation scans	*h* = −5→5
Absorption correction: multi-scan (*SADABS*; Bruker, 2007)	*k* = −11→11
*T*_min_ = 0.859, *T*_max_ = 0.977	*l* = −17→17
4255 measured reflections	

### Refinement

**Table:**

Refinement on *F*^2^	Primary atom site location: structure-invariant direct methods
Least-squares matrix: full	Secondary atom site location: difference Fourier map
*R*[*F*^2^ > 2σ(*F*^2^)] = 0.034	Hydrogen site location: inferred from neighbouring sites
*wR*(*F*^2^) = 0.090	H atoms treated by a mixture of independent and constrained refinement
*S* = 1.04	*w* = 1/[σ^2^(*F*_o_^2^) + (0.0478*P*)^2^ + 0.1317*P*] where *P* = (*F*_o_^2^ + 2*F*_c_^2^)/3
1190 reflections	(Δ/σ)_max_ < 0.001
82 parameters	Δρ_max_ = 0.30 e Å^−^^3^
3 restraints	Δρ_min_ = −0.22 e Å^−^^3^

### Special details

**Table:**

Geometry. All e.s.d.'s (except the e.s.d. in the dihedral angle between two l.s. planes) are estimated using the full covariance matrix. The cell e.s.d.'s are taken into account individually in the estimation of e.s.d.'s in distances, angles and torsion angles; correlations between e.s.d.'s in cell parameters are only used when they are defined by crystal symmetry. An approximate (isotropic) treatment of cell e.s.d.'s is used for estimating e.s.d.'s involving l.s. planes.
Refinement. Refinement of *F*^2^ against ALL reflections. The weighted *R*-factor *wR* and goodness of fit *S* are based on *F*^2^, conventional *R*-factors *R* are based on *F*, with *F* set to zero for negative *F*^2^. The threshold expression of *F*^2^ > 2σ(*F*^2^) is used only for calculating *R*-factors(gt) *etc*. and is not relevant to the choice of reflections for refinement. *R*-factors based on *F*^2^ are statistically about twice as large as those based on *F*, and *R*- factors based on ALL data will be even larger.

### Fractional atomic coordinates and isotropic or equivalent isotropic displacement parameters (Å^2^)

**Table:**

	*x*	*y*	*z*	*U*_iso_*/*U*_eq_	
C1	0.1936 (3)	0.09488 (12)	0.31650 (8)	0.0164 (2)	
C2	0.2433 (3)	−0.01250 (13)	0.23807 (8)	0.0206 (3)	
H21	0.165 (3)	−0.1064 (14)	0.2306 (11)	0.025*	
C3	−0.0658 (3)	−0.10152 (12)	0.41721 (8)	0.0168 (2)	
H31	0.016 (3)	−0.1708 (14)	0.3686 (10)	0.020*	
H32	−0.308 (3)	−0.1005 (15)	0.4067 (10)	0.020*	
C4	−0.0370 (3)	0.15403 (11)	0.47597 (8)	0.0169 (2)	
H41	−0.2791	0.1688	0.4685	0.020*	
H42	0.0701	0.2495	0.4638	0.020*	
N1	0.0575 (2)	0.04673 (10)	0.40139 (7)	0.0165 (2)	
N2	0.4038 (2)	0.03769 (10)	0.16317 (7)	0.0208 (2)	
N3	0.5432 (3)	0.08327 (13)	0.10006 (8)	0.0295 (3)	
O1	0.2787 (2)	0.22436 (9)	0.30680 (6)	0.0208 (2)	

### Atomic displacement parameters (Å^2^)

**Table:**

	*U*^11^	*U*^22^	*U*^33^	*U*^12^	*U*^13^	*U*^23^
C1	0.0158 (5)	0.0172 (5)	0.0158 (5)	0.0018 (4)	−0.0009 (4)	0.0016 (4)
C2	0.0285 (6)	0.0176 (5)	0.0162 (5)	−0.0020 (4)	0.0047 (4)	0.0010 (4)
C3	0.0204 (5)	0.0141 (5)	0.0162 (5)	−0.0021 (4)	0.0026 (4)	−0.0006 (4)
C4	0.0202 (5)	0.0142 (5)	0.0167 (5)	0.0009 (4)	0.0030 (4)	−0.0012 (4)
N1	0.0221 (4)	0.0131 (4)	0.0145 (4)	−0.0008 (3)	0.0035 (3)	−0.0006 (3)
N2	0.0264 (5)	0.0189 (5)	0.0171 (5)	0.0024 (4)	0.0021 (4)	−0.0006 (4)
N3	0.0395 (6)	0.0282 (6)	0.0222 (5)	−0.0010 (5)	0.0110 (5)	0.0007 (4)
O1	0.0282 (4)	0.0154 (4)	0.0193 (4)	−0.0016 (3)	0.0047 (3)	0.0017 (3)

### Geometric parameters (Å, º)

**Table:**

C1—O1	1.2367 (14)	C3—H31	0.978 (12)
C1—N1	1.3621 (13)	C3—H32	0.985 (12)
C1—C2	1.4504 (16)	C4—N1	1.4602 (13)
C2—N2	1.3099 (14)	C4—H41	0.9900
C2—H21	0.915 (12)	C4—H42	0.9900
C3—N1	1.4589 (13)	N2—N3	1.1239 (14)
C3—C4^i^	1.5193 (15)		
			
O1—C1—N1	121.76 (10)	H31—C3—H32	107.6 (11)
O1—C1—C2	120.75 (10)	N1—C4—C3^i^	110.54 (9)
N1—C1—C2	117.48 (10)	N1—C4—H41	109.5
N2—C2—C1	114.31 (10)	C3^i^—C4—H41	109.5
N2—C2—H21	115.7 (9)	N1—C4—H42	109.5
C1—C2—H21	129.7 (9)	C3^i^—C4—H42	109.5
N1—C3—C4^i^	110.57 (9)	H41—C4—H42	108.1
N1—C3—H31	111.3 (8)	C1—N1—C3	125.53 (9)
C4^i^—C3—H31	109.1 (8)	C1—N1—C4	119.16 (9)
N1—C3—H32	108.8 (8)	C3—N1—C4	114.17 (8)
C4^i^—C3—H32	109.5 (8)	N3—N2—C2	178.58 (12)
			
O1—C1—C2—N2	4.86 (16)	C2—C1—N1—C4	−171.95 (9)
N1—C1—C2—N2	−173.69 (9)	C4^i^—C3—N1—C1	137.76 (10)
O1—C1—N1—C3	176.58 (10)	C4^i^—C3—N1—C4	−54.61 (12)
C2—C1—N1—C3	−4.88 (15)	C3^i^—C4—N1—C1	−136.92 (9)
O1—C1—N1—C4	9.51 (15)	C3^i^—C4—N1—C3	54.59 (12)

Symmetry code: (i) −*x*, −*y*, −*z*+1.

### Hydrogen-bond geometry (Å, º)

**Table:**

*D*—H···*A*	*D*—H	H···*A*	*D*···*A*	*D*—H···*A*
C2—H21···O1^ii^	0.92 (1)	2.39 (1)	3.2219 (15)	151 (1)

Symmetry code: (ii) −*x*, *y*−1/2, −*z*+1/2.
